# Effect of lipid source and emulsifier on productive and physiological parameters of broilers

**DOI:** 10.5713/ab.20.0621

**Published:** 2020-12-11

**Authors:** Karine Isabela Tenório, Cinthia Eyng, Cristiane Regina do Amaral Duarte, Ricardo Vianna Nunes, Jomara Broch, Nilton Rohloff Júnior, Tânia Luiza Köhler, Edinan Hagdon Cirilo

**Affiliations:** 1 Department of Animal Science, Western Paraná State University, Unioeste, Marechal Cândido Rondon, PR, 85960-000, Brazil; 2 Department of Biological Sciences, State University of Mato Grosso, Unemat, Tangará da Serra, MT, 78300-000, Brazil

**Keywords:** Acid Soybean Oil, Degummed Soybean Oil, Exogenous Emulsifier, Ileal Digestibility, Pancreatic Lipase

## Abstract

**Objective:**

This study aimed to evaluate the replacement of degummed soybean oil (DSO) by acid soybean oil (ASO) in diets with or without the inclusion of emulsifier on broiler performance, relative organ weight, lipase activity, intestinal morphometry, and nutrient digestibility.

**Methods:**

A total of 704 1-day-old male broiler chicks were allotted to a 2×2 completely randomized factorial design (with or without emulsifier × two lipid sources [ASO and DSO]), with eight replicates and 22 birds each. The metabolizable energy level in diets with emulsifier was reduced by 40 kcal/kg from 1 to 21 d and 50 kcal/kg from 22 to 49 d.

**Results:**

Broilers fed diets containing ASO without emulsifier had higher (p = 0.005) weight gain than DSO-fed animals and with the inclusion of emulsifier had worse (p = 0.018) feed conversion ratio (FCR). Birds fed diets with emulsifier worsened FCR regardless of lipid source from 1 to 21 days (p = 0.006) and from 1 to 49 days (p = 0.0002). There was an increase (p = 0.026) in the relative pancreas weight, at 14 days, in birds fed diets containing ASO. Lipase activity and morphometry of the duodenum and jejunum, at 14 and 21 days, were not affected (p>0.05). The dietary inclusion of emulsifier improved the digestible energy (p = 0.053) in the presence of ASO. For the digestibility coefficients (gross energy, crude protein, and mineral matter), no interference was observed (p>0.05).

**Conclusion:**

The inclusion of emulsifier to energy-restricted diet with ASO maintained broiler performance in the first week, but worsened FCR in subsequent phases. The ASO can be considered as an alternative lipid source to DSO and does not interfere with the morphophysiological characteristics and performance of broilers. The combination of ASO and emulsifier increased the digestible energy content by 6.2%.

## INTRODUCTION

The inclusion of lipids in poultry diets is essential to meet energy requirements and achieve high animal performance. Lipid sources are added to diets at an average level of 3% in the starter phase, reaching up to 7% in the finisher phase [1]. Degummed soybean oil (DSO), the main lipid source used in the industry, is expensive, and therefore, acid soybean oil (ASO), which is a residue obtained from the neutralization of soybean oil for human consumption, has aroused the interest of the livestock industry [2]. This by-product has been considered an adequate energy source (7,153 kcal/kg dry matter [DM] of metabolizable energy) compared to soybean oil (8,825 kcal/kg DM of metabolizable energy) [1,3], and based on the price in Brazil (September/2020), the cost of ASO may be 37.1% lower than of DSO.

However, the chemical structure of fatty acids in ASO differs from that of fatty acids in soybean oil. Compared to DSO, the ASO presents low percentages of linoleic acid, linolenic acid, and total polyunsaturated fatty acids and a higher amount of total monounsaturated fatty acids and oleic acid [2]. In addition, this by-product has a high content of free fatty acids, which can directly affect its nutritional value. The high content of free fatty acids is correlated with decreased energy use due to reduced micellar affinity, thus interfering with the absorption process. Moreover, free fatty acids have a high capacity to form insoluble soaps with divalent minerals [4].

Broiler age is another factor that interferes with lipid use. The ability to digest lipids in broilers, especially in the starter phase, is limited by low lipase synthesis and low production of bile salts [5]. Therefore, the dietary inclusion of an emulsifying agent could be used as a strategy to compensate for the differences in the chemical characteristics of lipid sources and to overcome the physiological limitations of birds. Emulsifiers reduce the surface tension of lipid droplets and allow physical agitation by the gastrointestinal tract to break them into smaller particles, thus increasing lipid digestion. Moreover, emulsifiers are involved in micelle formation, which facilitates the absorption of dietary lipids. Studies show that a glyceryl polyethyleneglycol ricinoleate-based emulsifier improves the performance and lipid digestibility in poultry and pigs. Roy et al [6] found that the dietary inclusion of emulsifier at a dose of 1% of added fat (saturated palm oil) increased the live weight of broilers by approximately 5% at an age of 39 days. Kaczmarek et al [7] observed that dietary supplementation of emulsifier at a concentration of 0.04% caused a higher apparent total tract digestibility of ether extract by 5.4% and 4.8% for broilers at d 14 and 35, respectively. In addition, Udomprasert and Rukkwamsuk [8] observed an improvement in average daily gain of 19.65 gm/d and 0.06 in the feed conversion ratio (FCR) of pigs fed diets containing 5% of crude soybean oil and 1% of emulsifier.

We hypothesize that ASO is an alternative to DSO in diets for broilers. Furthermore, the dietary inclusion of emulsifier would favor the digestion of these lipid sources, providing energy and enhancing broiler performance, especially in the starter phase, allowing the possibility to reduce energy in poultry diets. Thus, we evaluated the effect of replacing DSO with ASO in diets reduced in metabolizable energy with the inclusion of a glyceryl polyethyleneglycol ricinoleate-based emulsifier and in diets without the additive and meeting the energy requirement on broiler performance, relative organ weight, lipase activity, intestinal morphometry, and nutrient digestibility.

## MATERIALS AND METHODS

### Animal care

The procedures involving animals were previously approved by the Ethics and Biosafety Committee of the institution (protocol #55/19). The broilers were raised according to the ethical guidelines for animal research established by the National Council for Animal Experimentation Control (CONCEA).

### Housing, birds, and treatments

The study was carried out at the Poultry Research Center of the Western Paraná State University - Unioeste, Marechal Cândido Rondon, Paraná, Brazil.

A total of 704 1-day-old Ross 308 AP male broiler chicks with an average initial body weight of 44.04±0.4 g from 45-week-old breeder hens were used. The birds were allotted to a 2×2 completely randomized factorial design (with or without the inclusion of emulsifier × two lipid sources [ASO and DSO]), with eight replicates and 22 birds per experimental unit (EU). The birds were housed in 1.76 m2 boxes provided with clean wood shaving bedding and food and water *ad libitum*. The lighting program and environment temperature were set as recommended by the Ross 308 AP manual. During the starter period, the environment was heated using electric brooders (250 watts) and circulation fans. If necessary, the environment was cooled using exhaust fans and evaporative cooling pads.

The experimental diets were formulated based on the chemical composition of feed ingredients and the nutritional requirements as proposed by Rostagno et al [1] for intermediate-performance male broilers from 1 to 7, 8 to 21, 22 to 33, 34 to 42, and 43 to 49 days of age (Tables 1 to 3). The glyceryl polyethyleneglycol ricinoleate-based emulsifier was added to diets at 350 g/ton, according to the manufacturer’s recommendation. The recommended metabolizable energy level was reduced by 40 kcal/kg from 1 to 21 days and 50 kcal/kg from 22 to 49 days of age in diets containing the emulsifying agent.

### Broiler performance

Body weight and feed intake of birds of each EU were recorded at seven, 21, 35, and 49 days of age to assess broiler performance (weight gain [WG], average feed intake, and FCR). Mortality was recorded daily for calculation of mortality-adjusted WG according to Sakomura and Rostagno [9].

### Relative weight of organs and lipase activity

At 14 and 21 days of age, one representative bird per EU (mean weight ±5%) was selected and slaughtered by cervical dislocation. The liver and pancreas were collected and weighed to calculate the relative organ weight (organ to live body weight ratio). Subsequently, the pancreas was frozen at −20°C for further analysis of pancreatic lipase activity. For this, the pancreas was homogenized using an IKA ultra-Turrax homogenizer (1:20 g/mL) in 50 mM Tris-HCl buffer solution (pH 8) containing 50 mM CaCl_2_ [10]. Lipase activity was obtained using 2,3-dimercaptopropanol tributyrate (BALB) as substrate and dithiobis-2-nitrobenzoic acid (DTNB) as chromophore (BALB-DNTP method, Gold Analisa, Belo Horizonte, MG, Brazil). Enzyme activity was expressed as international units (IU) per milligram of protein, according to Bradford’s method [11].

### Intestinal morphology

For morphological analyses, fragments of approximately 5 cm in length of the duodenum and jejunum of birds slaughtered at 14 and 21 days were taken to evaluate villus height, crypt depth, villus height to crypt depth ratio, and absorptive area. For sampling, the small intestine segments were divided into duodenum (from pylorus to distal duodenal loop) and jejunum (from distal duodenal loop to Meckel’s diverticulum). The fragments were fixed in buffered formalin solution (10%), dehydrated with a graded alcohol series, and embedded in paraffin. The 5-μm thick sections of each segment were mounted on glass slides and stained with hematoxylin and eosin, according to Luna [12]. Measurements were performed using the PROPLUS image system 4.1. Thirty villi and crypts were measured in each sample to evaluate villus height, villus width, crypt width, and crypt depth. The morphometric measurements were used to calculate the absorptive surface area of the intestinal mucosa according to the formula proposed by Kisielinski et al [13]: Absorption area = ([WV×HV]+[WV/2+WC/2]^2^ –[WV/2]^2^)/([WV/2+WC/2]^2^), where WV is the width of the villus, HV is the height of the villus, and WC is the width of the crypt. The villus height to crypt depth ratio was also calculated.

### Ileal digestibility

Nutrient digestibility and digestible energy (DE) content were determined using the ileal digestibility method in broilers at 49 days of age. A source of silica, Celite (Lompoc, CA, USA), was added to all experimental diets as an indigestible marker at 1% for 7 days. After adaptation to the diets, two representative birds per EU (mean weight ±5%) were individually weighed and slaughtered by cervical dislocation. Ileal digesta from the Meckel’s diverticulum to approximately 4 cm proximal to the ileocecal junction were collected. Samples of ileal digesta were homogenized and immediately oven-dried at 55°C for 72 hours. After drying, ileal digesta samples and experimental diets were ground and sent to the laboratory for analysis of DM, ash, crude protein (CP), gross energy (GE), and acid-insoluble ash (AIA). The DM, ash, and CP analyzed by the Kjeldahl method, were determined according to the AOAC [14]. The samples were pelleted and combusted in a bomb calorimeter for determination of GE. The AIA was determined according to the method of Van Keulen and Young [15]. Then, the digestibility coefficients (DC) of DM, ash, and CP, as well as the DE values were calculated [9].

### Statistical analysis

Data normality was evaluated using the Shapiro Wilk test. The data were submitted to analysis of variance using the SAS Software (SAS Inst. Inc., Cary, NC, USA). When significant, the factors were compared using the F-test at a significance level of 5%.

## RESULTS

There was a significant interaction between lipid sources and dietary inclusion of emulsifier on WG (p = 0.018) and FCR (p = 0.046) of broilers from 1 to 35 days of age. The unfolding of interaction showed that broilers fed diets containing ASO without emulsifier had higher WG than DSO-fed animals (2,520 g vs 2,412 g). Moreover, broilers fed ASO-based diets with the inclusion of emulsifier had worse FCR than birds receiving diets with DSO plus emulsifier (1.491 vs 1.473) and than those receiving the same lipid source but without the emulsifying agent (1.491 vs 1.450). The analysis of isolated factors showed that the FCR of birds fed diets with emulsifier worsened regardless of lipid source from 1 to 21 days (p = 0.006) and from 1 to 49 days (p = 0.0002) (Table 4).

No interaction (p>0.05) was observed between the studied factors on liver and pancreas relative weights at 14 and 21 days of age. However, birds fed diets containing ASO had higher (p = 0.026) relative pancreas weight at 14 days of age than DSO-fed animals, regardless of dietary inclusion of emulsifier (0.45% vs 0.41%, Table 5). There was no interaction (p>0.05) between lipid source and emulsifier inclusion on lipase activity at 14 and 21 days of age. Despite the influence of lipid source on the relative pancreas weight at 14 days of age, no significant effect of isolated factors (p>0.05) was observed for lipase activity (Table 6).

Morphometric parameters (villus height, crypt depth, villus height to crypt depth ratio, and absorptive area) of the duodenum and jejunum segments at 14 and 21 days were not influenced (p>0.05) by lipid sources and emulsifier inclusion (Tables 7, 8).

There was an interaction (p = 0.053) between lipid source and emulsifier inclusion on DE content. The analysis within each lipid source showed that the emulsifier led to better use of the ASO, resulting in higher DE (3,084 kcal/kg vs 2,904 kcal/kg). The dietary inclusion of emulsifier led to higher DE for ASO than DSO (3,084 kcal/kg vs 2,847 kcal/kg). No effect of treatments (p>0.05) was observed for nutrient DCs (Table 9).

## DISCUSSION

Although ASO is considered a source with reduced use by broilers due to its high content of free fatty acids, the dietary inclusion of this alternative lipid source improved the WG of broilers from 1 to 35 days of age and did not impair their morphophysiological characteristics. Rodriguez-Sanchez et al [4] reported higher WG at 21 days in broilers fed diets with 15% of free fatty acids. In this study, the DE values of ASO and DSO in diets without the emulsifying agent were similar, as also observed by Rodrigues-Sanchez et al [4]. These authors suggest that this result may be related to the presence of free fatty acids in the diet, which are the final products of lipid digestion and do not influence the activity of pancreatic lipase. Moreover, free fatty acids participate in triglyceride emulsification [16] and induce the formation of a high-affinity complex between lipase and colipase [17], which increases the hydrolytic activity of lipase. In addition, the difference in the chemical structure of fatty acids of the lipid sources used in this trial was lower than expected considering the data reported in the literature [2], which may have favored the use by the birds. The ASO presented 47.5% of linoleic acid, 4.6% of linolenic acid, and 28.5% of oleic acid compared to 53.1%, 7%, and 24.7% of DSO, respectively. Borsatti et al [3] stated that ASO, besides being an adequate energy source for broilers, reduces production costs and can be used as an alternative to DSO in the poultry industry.

Given that the morphophysiological changes in broilers are most pronounced during the starter phase (1 to 21 days), especially in relation to lipase activity and secretion of bile salts [5,18], we decided to evaluate the effects of lipid sources and emulsifier on organ weight, lipase activity in the pancreas, and intestinal morphometry at 14 and 21 days of age. Despite evidence that the studied factors may interfere with the above-mentioned parameters [19,20], no changes were observed in our study, except for the influence of lipid source on relative pancreas weight at 14 days of age.

Feeding lipid sources may change pancreatic lipase activity [21], and pancreas weight is an indirect measure of pancreatic activity as it is directly associated with endogenous enzyme production. Sakomura et al [21] report that the growth of the pancreas coincided with an increase in the production of digestive enzymes. The ASO has a lower digestibility than DSO [1,4] due to its fatty acid profile. Therefore, the higher relative pancreas weight could be related to the higher demand for lipase. However, this relationship was not observed in this study as the different lipid sources did not affect lipase activity in the pancreas.

The emulsifier used in this study (glyceryl polyethyleneglycol ricinoleate) is extracted from an oil source and acts as a feed additive, i.e., it helps increase energy supply to poultry [7,22]. During the first week of the experiment, inclusion of the emulsifier compensated for the 40 kcal/kg decrease in apparent metabolizable energy requirement of birds, regardless of the lipid source used, since performance was similar between groups fed diets with and without the emulsifier. After this period, the influence of the emulsifier on the processes of lipid digestion and absorption was not able to compensate for the decrease in dietary energy, which resulted in a worse FCR.

Exogenous emulsifiers, as well as bile salts, form a bridge between water- and fat-soluble materials and increase the active surface of fats [23]. As a result, emulsifying agents enhance the action of lipase, which helps to hydrolyze triglyceride molecules into fatty acids and monoglycerides [24] and favors the formation of micelles consisting of lipolysis products. Thus, the additive may have been effective in influencing the processes of lipid digestion and absorption during the first week of life because starter chicks have a physiological limitation regarding the production of bile salts and endogenous lipase [5]. The fact that the emulsifying agent did not affect lipase activity in the pancreas can lead to the hypothesis that this additive influences the processes of lipid digestion and absorption, such as facilitating micelle formation, indirectly enhancing the activity of lipase in the gastrointestinal tract. The energy values of DSO for broilers increased up to 21 days of age, as reported by Bertechini et al [25], which indicates the development of the digestive system up to 21 days and improvement in lipid use. Khonyoung et al [26] reported that feeding lysolecithin as an emulsifier improved broiler performance up to 21 days of age, which demonstrates that the addition of an emulsifier to broiler diets would improve the use of dietary lipids up to 21 days of age.

Given the results on performance and digestibility, it is essential to highlight that the difference in the chemical structure of the fatty acids present in the lipid sources did not interfere with lipid use by the birds, despite the influence of the emulsifier on the DE content. The inclusion of the emulsifier in diets with ASO increased the DE content by 6.2%. The ASO is a residue obtained from the neutralization of soybean oil for human consumption. It corresponds to approximately 6% of the raw material and is composed of a mixture of free fatty acids, mono-, and diglycerides [27]. The high concentration of free fatty acids is correlated with reduced digestibility due to an inefficient emulsification process, which negatively interferes with micelle formation and, consequently, lipid absorption [3,27]. The dietary inclusion of emulsifiers is expected to increase the digestibility of lipid sources by promoting the incorporation of fatty acids into micelles, especially free fatty acids [28]. In this study, broilers fed diets containing ASO and the emulsifier had a higher DE than those fed DSO-based diets. However, the beneficial effect of the emulsifying agent could not counterbalance the feed conversion of broilers fed diets without nutrient reduction. This reduction may have been overestimated given the expected energy supply capacity of the additive. In a study evaluating the inclusion of an emulsifier similar to that used in our study in diets for broilers, Kulkarni et al [29] observed a positive effect of the emulsifying agent on feed conversion and metabolizable energy. However, the additive was top-dressed and was not considered in the nutritional matrix.

In conclusion, the addition of a glyceryl polyethyleneglycol ricinoleate-based emulsifier to diets with ASO and energy restriction maintained broiler performance in the first week of life, but worsened feed conversion in subsequent phases. ASO can be considered as an alternative lipid source to DSO and does not interfere with the morphophysiological characteristics and performance of broilers. The combination of ASO and emulsifier increased the DE content by 6.2%.

## Figures and Tables

**Table 1:** Ingredients and chemical composition of experimental diets containing different lipid sources, with or without inclusion of emulsifier, for the pre-starter (1 to 7 days) and starter (8 to 21 days) phases of broilers

Items	1 to 7 days				8 to 21 days			
	
	ASO		DSO		ASO		DSO	
			
	With	Without	With	Without	With	Without	With	Without
Ingredients (%)								
Corn	55.96	57.15	56.62	57.55	57.41	58.60	58.31	59.26
Soybean meal, 46%	34.40	34.20	34.30	34.10	32.30	32.10	32.20	32.00
Meat and bone meal, 45%	3.00	3.00	3.00	3.00	3.00	3.00	3.00	3.00
ASO	2.18	1.18	-	-	3.18	2.18	-	-
DSO	-	-	1.62	0.88	-	-	2.37	1.62
Poultry fat	1.00	1.00	1.00	1.00	1.00	1.00	1.00	1.00
Dicalcium phosphate	1.097	1.096	1.097	1.095	0.878	0.877	0.877	0.876
Limestone	0.598	0.600	0.599	0.600	0.512	0.514	0.513	0.515
NaCl	0.479	0.479	0.479	0.479	0.462	0.462	0.462	0.462
Mineral supplement^1)^	0.050	0.050	0.050	0.050	0.050	0.050	0.050	0.050
Vitamin supplement^2)^	0.130	0.130	0.130	0.130	0.130	0.130	0.130	0.130
DL-methionine, 99%	0.397	0.396	0.396	0.395	0.378	0.377	0.377	0.376
L-threonine, 98%	0.126	0.126	0.126	0.126	0.120	0.120	0.120	0.120
L-lysine HCL, 50.7%	0.489	0.495	0.492	0.496	0.488	0.494	0.493	0.497
Choline chloride, 60%	0.060	0.060	0.060	0.060	0.060	0.060	0.060	0.060
Inert^3)^	0.035	0.035	0.035	0.035	0.035	0.035	0.035	0.035
ME (kcal/kg)	2,975	2,935	2,975	2,935	3,050	3,010	3,050	3,010
Crude protein (%)	21.94	21.94	21.94	21.94	21.09	21.09	21.09	21.09
Calcium (%)	0.971	0.971	0.971	0.971	0.878	0.878	0.878	0.878
Avaiable phosphorus (%)	0.463	0.463	0.463	0.463	0.419	0.419	0.419	0.419
Sodium (%)	0.225	0.225	0.225	0.225	0.218	0.218	0.218	0.218
Digestible Lys (%)	1.307	1.307	1.307	1.307	1.256	1.256	1.256	1.256
Digestible Met+cys (%)	0.967	0.967	0.967	0.967	0.929	0.929	0.929	0.929
Digestible Thr (%)	0.863	0.863	0.863	0.863	0.829	0.829	0.829	0.829

ASO, acid soybean oil; DSO, degummed soybean oil; ME, metabolizable energy.

1) Mineral supplement, per kg of diet: 50 mg iron; 10 mg copper; 65 mg manganese; 65 mg zinc; 1 mg iodine.

2) Vitamin supplement, per kg of diet: 14,300 IU vitamin A; 5,200 IU vitamin D_3_; 71.5 IU vitamin E; 3.9 mg vitamin K_3_; 2.99 mg vitamin B_1_; 9.10 mg vitamin B_2_; 15.6 mg pantothenic acid; 5.2 mg vitamin B_6_; 3.25 mcg vitamin B_12_; 78 mg nicotinic acid; 2.6 mg folic acid; 325 mcg biotin; 390 mcg selenium.

3) The inclusion of the emulsifier was performed in substitution to the inert Kaolin = mineral kaolinite (São Carlos, SP, Brazil).

**Table 2:** Ingredients and chemical composition of experimental diets containing different lipid sources, with or without inclusion of emulsifier, for the grower I (22 to 33 days) and grower II (34 to 42 days) phases of broilers

Items	22 to 33 days				34 to 42 days			
	
	ASO		DSO		ASO		DSO	
			
	With	Without	With	Without	With	Without	With	Without
Ingredients (%)								
Corn	63.80	65.25	64.91	66.04	69.67	71.13	70.56	71.70
Soybean meal, 46%	25.90	25.70	25.70	25.50	21.00	20.80	20.90	20.70
Meat and bone meal, 45%	3.00	3.00	3.00	3.00	3.00	3.00	3.00	3.00
ASO	3.57	2.31	-	-	3.10	1.83	-	-
DSO	-	-	2.66	1.72	-	-	2.30	1.36
Poultry fat	1.00	1.00	1.00	1.00	1.00	1.00	1.00	1.00
Dicalcium phosphate	0.687	0.685	0.686	0.684	0.302	0.300	0.301	0.299
Limestone	0.374	0.377	0.376	0.378	0.337	0.340	0.339	0.341
NaCl	0.439	0.438	0.438	0.438	0.412	0.412	0.412	0.412
Mineral supplement^1)^	0.050	0.050	0.050	0.050	0.050	0.050	0.050	0.050
Vitamin supplement^2)^	0.100	0.100	0.100	0.100	0.100	0.100	0.100	0.100
DL-methionine, 99%	0.335	0.333	0.333	0.332	0.290	0.289	0.289	0.288
L-threonine, 98%	0.117	0.117	0.117	0.117	0.104	0.104	0.104	0.104
L-lysine HCL, 50.7%	0.534	0.541	0.539	0.544	0.545	0.552	0.550	0.555
Choline chloride, 60%	0.060	0.060	0.060	0.060	0.060	0.060	0.060	0.060
Inert^3)^	0.035	0.035	0.035	0.035	0.035	0.035	0.035	0.035
ME (kcal/kg)	3,150	3,100	3,150	3,100	3,200	3,150	3,200	3,150
Crude protein (%)	18.61	18.61	18.61	18.61	16.79	16.79	16.79	16.79
Calcium (%)	0.758	0.758	0.758	0.758	0.634	0.634	0.634	0.634
Avaiable phosphorus (%)	0.374	0.374	0.374	0.374	0.296	0.296	0.296	0.296
Sodium (%)	0.208	0.208	0.208	0.208	0.197	0.197	0.197	0.197
Digestible Lys (%)	1.124	1.124	1.124	1.124	1.014	1.014	1.014	1.014
Digestible Met+cys (%)	0.832	0.832	0.832	0.832	0.750	0.750	0.750	0.750
Digestible Thr (%)	0.742	0.742	0.742	0.742	0.669	0.669	0.669	0.669

ASO, acid soybean oil; DSO, degummed soybean oil; ME, metabolizable energy.

1) Mineral supplement, per kg of diet: 50 mg iron; 10 mg copper; 65 mg manganese; 65 mg zinc; 1 mg iodine.

2) Vitamin supplement, per kg of diet: 11,000 IU vitamin A; 4,000 IU vitamin D_3_; 55 IU vitamin E; 3 mg vitamin K_3_; 2.3 mg vitamin B_1_; 7 mg vitamin B_2_; 12 mg pantothenic acid; 4 mg vitamin B_6_; 2.5 mcg vitamin B_12_; 60 mg nicotinic acid; 2 mg folic acid; 250 mcg biotin; 300 mcg selenium.

3) The inclusion of the emulsifier was performed in substitution to the inert Kaolin = mineral kaolinite (São Carlos, SP, Brazil).

**Table 3:** Ingredients and chemical composition of experimental diets containing different lipid sources with or without inclusion of emulsifier for the finisher (42 to 49 days) phase of broilers

Items	ASO		DSO	
	
	With	Without	With	Without
Ingredients (%)				
Corn	69.68	71.22	71.09	72.12
Soybean meal, 46%	18.90	18.60	18.60	18.50
Meat and bone meal, 45%	3.00	3.00	3.00	3.00
ASO	4.39	3.14	-	-
DSO	-	-	3.27	2.34
Poultry fat	1.00	1.00	1.00	1.00
Dicalcium phosphate	0.191	0.189	0.189	0.188
Limestone	0.289	0.292	0.291	0.293
NaCl	0.400	0.400	0.400	0.400
Mineral supplement^1)^	0.050	0.050	0.050	0.050
Vitamin supplement^2)^	0.100	0.100	0.100	0.100
DL-methionine, 99%	0.270	0.269	0.269	0.268
L-threonine, 98%	0.099	0.099	0.099	0.099
L-lysine HCL, 51%	0.537	0.544	0.543	0.549
Choline chloride, 60%	0.060	0.060	0.060	0.060
Inert^3)^	0.035	0.035	0.035	0.035
Celite	1.000	1.000	1.000	1.000
ME (kcal/kg)	3,250	3,200	3,250	3,200
Crude protein (%)	15.79	15.79	15.79	15.79
Calcium (%)	0.581	0.581	0.581	0.581
Avaiable phosphorus (%)	0.271	0.271	0.271	0.271
Sodium (%)	0.192	0.192	0.192	0.192
Digestible Lys (%)	0.954	0.954	0.954	0.954
Digestible Met+cys (%)	0.706	0.706	0.706	0.706
Digestible Thr (%)	0.630	0.630	0.630	0.630

ASO, acid soybean oil; DSO, degummed soybean oil; ME, metabolizable energy.

1) Mineral supplement, per kg of diet: 50 mg iron; 10 mg copper; 65 mg manganese; 65 mg zinc; 1 mg iodine.

2) Vitamin supplement, per kg of diet: 11,000 IU vitamin A; 4,000 IU vitamin D_3_; 55 IU vitamin E; 3 mg vitamin K_3_; 2.3 mg vitamin B_1_; 7 mg vitamin B_2_; 12 mg pantothenic acid; 4 mg vitamin B_6_; 2.5 mcg vitamin B_12_; 60 mg nicotinic acid; 2 mg folic acid; 250 mcg biotin; 300 mcg selenium.

3) The inclusion of the emulsifier was performed in substitution to the inert Caulim Kaolin = mineral kaolinite (São Carlos, SP, Brazil).

**Table 4:** Growth performance of broilers fed diets containing different lipid sources, with or without emulsifier

Items	Lipid source	1–7 days			1–21 days			1–35 days			1–49 days		
			
		WG	AFI	FCR	WG	AFI	FCR	WG	AFI	FCR	WG	AFI	FCR
Emulsifier													
+	ASO	150.69	170.34	1.131	1,003.03	1,292.97	1.289	2,450.58	3,653.77	1.491^Aa^	3,876.50	6,658.10	1.718
+	DSO	152.10	171.17	1.127	994.99	1,285.78	1.292	2,466.51	3,632.96	1.473^b^	3,837.95	6,585.79	1.717
−	ASO	149.44	166.34	1.114	1,020.09	1,291.63	1.266	2,520.49^a^	3,653.16	1.450^B^	3,994.59	6,692.70	1.676
−	DSO	150.34	171.76	1.144	1,002.54	1,277.44	1.274	2,412.24^b^	3,550.14	1.471	3,874.32	6,464.38	1.669
SEM		7.53	6.61	0.03	0.25	35.1	0.02	69.64	118.24	0.03	198.7	313.93	0.03
Emulsifier													
+		151.40	170.76	1.129	998.74	1,289.13	1.291	2,458.55	3,643.37	1.482	3,857.22	6,621.95	1.717
−		149.89	169.05	1.129	1,011.32	1,284.53	1.270	2,466.37	3,601.65	1.460	3,934.46	6,578.54	1.672
Lipid source													
ASO		150.07	168.34	1.122	1,011.56	1,292.30	1.278	2,485.54	3,653.47	1.470	3,935.54	6,675.40	1.697
DSO		151.22	171.47	1.135	998.51	1,281.89	1.284	2,439.38	3,591.55	1.472	3,856.14	6,525.09	1.693
CV (%)		5.00	3.89	3.06	2.47	2.73	1.43	2.83	3.20	1.84	5.10	4.76	4.78
p-value O×E		0.924	0.335	0.176	0.611	0.791	0.715	0.018	0.334	0.046	0.565	0.488	0.774
p-value oil (O)		0.668	0.192	0.305	0.177	0.421	0.400	0.071	0.150	0.856	0.268	0.187	0.715
p-value emulsifier (E)		0.577	0.472	0.997	0.194	0.714	0.006	0.753	0.327	0.032	0.281	0.699	0.0002

WG, weight gain (g); AFI, average feed intake (g); FCR, feed conversion rate (g/g); +, with emulsifier; −, without emulsifier; SEM, standard error of the mean; ASO, acid soybean oil; DSO, degummed soybean oil; CV, coefficient of variation.

A–B/a–b In the same column, means followed by uppercase letters or lowercase letters differed (p<0.05) regarding the type of lipid source or the inclusion or not of the emulsifier, respectively.

**Table 5:** Relative weight (%) of liver and pancreas of broilers fed diets containing different lipid sources, with or without emulsifier

Items	Lipid source	14 days		21 days	
	
		Liver	Pancreas	Liver	Pancreas
Emulsifier					
+	ASO	3.60	0.46	3.22	0.35
+	DSO	3.61	0.42	3.33	0.34
−	ASO	3.44	0.44	3.37	0.32
−	DSO	3.41	0.40	3.06	0.33
SEM		0.50	0.04	0.31	0.03
Emulsifier					
+		3.60	0.44^a^	3.28	0.34
−		3.42	0.42^b^	3.21	0.32
Lipid source					
ASO		3.52	0.45	3.29	0.34
DSO		3.51	0.41	3.19	0.33
CV (%)		14.21	10.39	9.54	10.12
p-value O×E		0.908	0.931	0.068	0.368
p-value oil (O)		0.952	0.026	0.363	0.623
p-value emulsifier (E)		0.328	0.220	0.556	0.103

+, with emulsifier; −, without emulsifier; ASO, acid soybean oil; DSO, degummed soybean oil; SEM, standard error of the mean; CV, coefficient of variation.

a,b In the same column, means followed by lowercase letters differed (p<0.05) regarding the type of lipid source.

**Table 6:** Lipase activity (UI/mg of protein) in pancreas of broilers fed diets containing different lipid sources, with or without emulsifier

Items	Lipid source	14 days	21 days
Emulsifier			
+	ASO	7.22	24.13
+	DSO	5.40	21.17
−	ASO	6.02	21.18
−	DSO	5.76	18.03
SEM		2.66	10.64
Emulsifier			
+		5.78	22.65
−		5.37	19.61
Lipid source			
ASO		6.11	22.66
DSO		5.04	19.60
CV (%)		47.72	50.35
p-value O×E		0.765	0.981
p-value oil (O)		0.270	0.423
p-value emulsifier (E)		0.625	0.425

+, with emulsifier; −, without emulsifier; ASO, acid soybean oil; DSO, degummed soybean oil; SEM, standard error of the mean; CV, coefficient of variation.

**Table 7:** Villus height, crypt depth, villus:crypt ratio, and area of absorption of duodenum of broilers fed diets containing different lipid sources, with or without emulsifier

Items	Lipid source	14 days				21 days			
	
		VH	CD	V:C	AA	VH	CD	V:C	AA
Emulsifier									
+	ASO	872.19	50.85	17.41	21.37	847.79	64.85	13.19	18.75
+	DSO	823.05	48.63	17.96	20.75	848.64	61.09	14.02	19.13
−	ASO	870.87	47.52	18.90	20.28	874.95	64.52	13.95	17.82
−	DSO	768.30	46.89	16.80	20.17	871.60	71.19	12.57	18.87
SEM		142.59	12.16	2.80	3.18	73.02	11.27	1.98	1.79
Emulsifier									
+		849.85	49.84	18.45	21.09	848.21	62.97	13.60	18.94
−		824.25	47.23	17.95	20.23	873.15	68.11	13.20	17.80
Lipid source									
ASO		871.53	49.18	18.16	20.83	861.37	64.69	13.57	18.29
DSO		795.67	47.76	18.22	20.46	861.00	66.52	13.24	18.46
CV (%)		17.03	25.06	15.40	15.41	8.48	17.17	17.76	9.74
p-value O×E		0.667	0.881	0.075	0.854	0.944	0.261	0.178	0.781
p-value oil (O)		0.230	0.787	0.546	0.793	0.966	0.751	0.730	0.815
p-value emulsifier (E)		0.652	0.632	0.498	0.546	0.402	0.292	0.667	0.136

VH, villus height (μm); CD, crypt depth (μm); V:C, villus:crypt ratio; AA, area of absorption (μm^2^); +, with emulsifier; −, without emulsifier; ASO, acid soybean oil; DSO, degummed soybean oil; SEM, standard error of the mean; CV, coefficient of variation.

**Table 8:** Villus height, crypt depth, villus:crypt ratio, and area of absorption of jejunum of broilers fed diets containing different lipid sources, with or without emulsifier

Items	Lipid source	14 days				21 days			
	
		VH	CD	V:C	AA	VH	CD	V:C	AA
Emulsifier									
+	ASO	332.29	38.17	8.72	8.66	410.87	47.20	8.70	9.81
+	DSO	383.45	45.04	8.73	9.05	379.26	46.84	8.12	8.34
−	ASO	374.06	41.61	9.12	9.40	422.05	49.88	8.48	8.93
−	DSO	347.58	39.92	8.88	8.82	455.01	55.61	8.34	9.73
SEM		62.68	8.46	1.43	1.19	83.75	9.03	0.82	1.56
Emulsifier									
+		344.18	41.60	8.72	8.85	395.07	47.02	8.41	9.08
−		361.70	40.82	9.01	9.13	422.87	49.65	8.41	9.36
Lipid source									
ASO		354.57	40.00	8.93	9.06	416.03	48.43	8.60	9.41
DSO		352.41	42.48	8.81	8.93	399.77	48.04	8.23	9.04
CV (%)		17.73	20.74	16.16	13.20	20.53	18.73	9.75	16.97
p-value O×E		0.284	0.191	0.818	0.282	0.619	0.989	0.500	0.072
p-value oil (O)		0.988	0.423	0.834	0.823	0.654	0.912	0.268	0.586
p-value emulsifier (E)		0.517	0.794	0.608	0.565	0.408	0.467	1.000	0.678

VH, villus height (μm); CD, crypt depth (μm); V:C, villus:crypt ratio; AA, area of absorption (μm^2^); +, with emulsifier; −, without emulsifier; ASO, acid soybean oil; DSO, degummed soybean oil; SEM, standard error of the mean; CV, coefficient of variation.

**Table 9:** Average values of digestible energy (kcal/kg) and digestibility coefficients (%) of dry matter, gross energy and crude protein determined in broilers at 49 days of age fed diets containing different lipid sources, with or without emulsifier

Items	Lipid source	DE	CDDM	CDGE	CDCP
Emulsifier					
+	ASO	3,084^Aa^	67.85	71.18	66.26
+	DSO	2,847^b^	67.51	69.48	69.79
−	ASO	2,904^B^	66.83	69.03	69.64
−	DSO	2,865	66.92	69.07	69.79
SEM		78.34	1.78	1.90	2.40
Emulsifier					
+		2,965	67.67	70.33	68.03
−		2,884	66.87	69.05	68.09
Lipid source					
ASO		2,994	67.34	70.10	67.95
DSO		2,856	67.21	69.27	68.17
CV (%)		3.00	2.93	3.02	6.38
p-value O×E		0.053	0.834	0.426	0.153
p-value oil (O)		0.008	0.896	0.446	0.922
p-value emulsifier (E)		0.090	0.432	0.247	0.979

DE, digestible energy, dry matter basis; CDDM, coefficient of digestibility of dry matter; CDGE, coefficient of digestibility of gross energy; CDCP, coefficient of digestibility of crude protein; +, with emulsifier; −, without emulsifier; ASO, acid soybean oil; DSO, degummed soybean oil; SEM, standard error of the mean; CV, coefficient of variation.

A–B/a–b In the same column, means followed by uppercase letters or lowercase letters differed (p<0.05) regarding the type of lipid source or the inclusion or not of the emulsifier, respectively.
